# Comparison of DCE‐MRI kinetic parameters and FMISO‐PET uptake parameters in head and neck cancer patients

**DOI:** 10.1002/mp.12228

**Published:** 2017-04-20

**Authors:** Urban Simoncic, Sara Leibfarth, Stefan Welz, Nina Schwenzer, Holger Schmidt, Gerald Reischl, Christina Pfannenberg, Christian la Fougère, Konstantin Nikolaou, Daniel Zips, Daniela Thorwarth

**Affiliations:** ^1^ Section for Biomedical PhysicsDepartment of Radiation OncologyUniversity Hospital TübingenTübingenGermany; ^2^ Faculty of Mathematics and PhysicsUniversity of LjubljanaLjubljanaSlovenia; ^3^ Jozef Stefan InstituteLjubljanaSlovenia; ^4^ Department of Radiation OncologyUniversity Hospital TübingenTübingenGermany; ^5^ Diagnostic and Interventional RadiologyDepartment of RadiologyUniversity Hospital TübingenTübingenGermany; ^6^ Preclinical Imaging and RadiopharmacyDepartment of RadiologyUniversity Hospital TübingenTübingenGermany; ^7^ Nuclear MedicineDepartment of RadiologyUniversity Hospital TübingenTübingenGermany

**Keywords:** DCE‐MRI, FMISO, hypoxia, image quantification, kinetic analysis, PET, vascular kinetic parameters

## Abstract

**Purpose:**

Tumor hypoxia is a major cause of radiation resistance, often present in various solid tumors. Dynamic [^18^F]‐fluoromisonidazole (FMISO) PET imaging is able to reliably assess tumor hypoxia. Comprehensive characterization of tumor microenvironment through FMISO‐PET and dynamic contrast enhanced (DCE) MR multimodality imaging might be a valuable alternative to the dynamic FMISO‐PET acquisition. The aim of this work was to explore the correlation between the FMISO‐PET and DCE‐MRI kinetic parameters.

**Methods:**

This study was done on head and neck cancer patients (N = 6), who were imaged dynamically with FMISO‐PET and DCE‐MRI on the same day. Images were registered and analyzed for kinetics on a voxel basis. FMISO‐PET images were analyzed with the two‐tissue compartment three rate‐constant model. Additionally, tumor‐to‐muscle ratio (TMR) maps were evaluated. DCE‐MRI was analyzed with the extended Tofts model. Voxel‐wise Pearson's coefficients were calculated for each patient to assess pairwise parameter correlations.

**Results:**

Median correlations between FMISO uptake parameters and DCE‐MRI kinetic parameters varied across the parameter pairs in the range from −0.05 to 0.71. The highest median correlation of r = 0.71 was observed for the pair *V*_*b*_−*v*_*p*_, while the *K*_*1*_−*K* ^*trans*^ median correlation was r = 0.45. Median correlation coefficients for the *K*_*1*_−*v*_*p*_ and the *K*_*i*_−*K* ^*trans*^ pairs were r = 0.42 and r = 0.32, respectively. Correlations between FMISO uptake rate parameter *K*_*i*_ and DCE‐MRI kinetic parameters varied substantially across the patients, whereas correlations between the FMISO and DCE‐MRI vascular parameters were consistently high. Median TMR‐*K*_*1*_ and TMR‐*K* ^*trans*^ correlations were r = 0.52 and r = 0.46, respectively, but varied substantially across the patients.

**Conclusions:**

Based on this clinical evidence, we can conclude that the vascular fraction parameters obtained through DCE‐MRI kinetic analysis or FMISO kinetic analysis measure the same biological property, while other kinetic parameters are unrelated. These results might be useful in the design of future clinical trials involving FMISO‐PET/DCE‐MR multimodality imaging for the assessment of tumor microenvironment.

## 1 Introduction

Tumor hypoxia is a well‐known phenomenon that is often present in various solid tumors. It has long been recognized as impairing response to radiotherapy (RT).^1^, ^2^, ^3^ In addition, tumor hypoxia also reduces the therapeutic effect for a number of chemotherapeutic agents,^4^, ^5^, ^6^ and it is anticipated that hypoxia affects the curability of solid tumors, regardless of the treatment modality.^7^ Tumor perfusion is another significant tumor microenvironment characteristic with potential clinical impact. Higher overall perfusion or lower skewness of the perfusion usually indicates favorable therapy response.^8^, ^9^, ^10^ Better perfused tumors may cause better tumor oxygenation and therefore weaker therapy resistance. However, hypoxia also stimulates angiogenesis.^11^ Therefore, increased tumor perfusion might be a consequence of the hypoxia and highly perfused tumors may be still hypoxic, whereas the tumor vascularity is irregular and apparent blood flow actually contains mainly plasma.^12^

One promising imaging modality for hypoxia assessment is positron emission tomography (PET) imaging using the hypoxia tracer [^18^F]‐fluoromisonidazole (FMISO).^13^, ^14^ A static FMISO‐PET scan acquired several hours post injection (p.i.) is often used as a measure of hypoxia,^15^, ^16^ but a dynamic imaging protocol that quantifies the perfusion and retention properties of FMISO may be superior.^17^ Such a dynamic PET imaging protocol is extremely complex, so it is not commonly used even in research. The dynamic contrast enhanced magnetic resonance imaging (DCE‐MRI), which uses contrast agents like gadolinium‐diethylenetriamine pentaacetic acid (Gd‐DTPA), is another imaging technique that provides perfusion and permeability information.^18^ Therefore, DCE‐MRI might provide similar information as the addition of dynamic PET acquisition to the FMISO‐PET imaging protocol.

Due to the efforts to precisely characterize tumors and their microenvironments, the use of multimodality imaging techniques is rapidly gaining acceptance in oncology.^19^ With the advent of combined PET/MRI scanners, the acquisition of intrinsically registered PET and MRI data has become possible.^20^ However, optimization of a FMISO‐PET/DCE‐MR multimodality imaging protocol for cancer patients would be much easier if the relations between FMISO‐PET and DCE‐MRI kinetic parameters were known. From the mechanisms of FMISO and Gd‐DTPA transport from the blood to the tissue, and the assumptions behind the models for kinetic analysis, one can expect highly correlated FMISO and DCE‐MRI vascular fractions. Parameters *K*_*1*_ and *K* ^*trans*^ are potentially correlated, while other correlations between the FMISO‐PET and DCE‐MRI parameters are not expected per se. In order to provide some of the missing knowledge for FMISO‐PET/DCE‐MR multimodality imaging protocol optimization, we assessed the correlation between the FMISO uptake parameters and DCE‐MRI kinetic parameters.

## 2 Methods and materials

### 2.A Subjects and imaging

The study was performed on a dataset of N = 6 head and neck cancer patients that were acquired prior to salvage RT. Patients were imaged with dynamic FMISO‐PET/CT and combined PET/MRI including DCE‐MRI on the same day, before the start of RT. The study was approved by the local ethics committee. All patients gave written informed consent for participating in the imaging study prior to any study‐related procedure.

#### 2.A.1 FMISO‐PET imaging

Patients were injected with FMISO (320–377 MBq, median: 349 MBq) simultaneously with the start of dynamic acquisition on the PET/CT scanner (Biograph mCT, Siemens Healthcare, Erlangen, Germany). FMISO‐PET image acquisitions were done in the RT treatment position with mask fixation. Dynamic PET data were framed as follows: 12 × 10 s, 8 × 15 s, 11 × 1 min and 5 × 5 min, resulting in a total duration of 40 min. Subsequently, two static PET/CT scans were acquired at 2 h p.i. and 4 h p.i. in the same anatomical position, with each acquisition lasting 15 min. Images were reconstructed to 45 slices, matrix size 200 × 200 and voxel size of 4.1 × 4.1 × 5.0 mm^3^, using the vendor‐provided OSEM 3D reconstruction algorithm with four iterations, eight subsets, and a 3D Gaussian filter of 5 mm.

#### 2.A.2 DCE‐MR imaging

Dynamic DCE‐MRI acquisition was performed on a combined 3 Tesla PET/MRI scanner (Biograph mMR, Siemens Healthcare, Erlangen, Germany) after an automatic fast bolus injection of 0.1 mmol Gd‐DTPA per kg of patient weight and a saline flush. The field of view included the entire tumor and the common carotid arteries. A total of 89 time frames were acquired with an axial view‐sharing T_1_‐weighted spoiled gradient echo sequence (TWIST) with the repetition time to echo time (TR/TE) equals to 2.86 ms/1.01 ms, flip angle 12°, temporal resolution 2.9 s, and bandwidth of 530 Hz/pixel. Images were reconstructed to 56 slices, matrix size 256 × 256, and voxel size of 1.1 × 1.1 × 4.0 mm^3^. For the derivation of the intrinsic tissue T_1_ relaxation times needed for mapping of a TWIST MRI signal intensities into contrast agent concentrations, additional volumetric interpolated breath‐hold examination (VIBE) sequences were acquired with two different flip angles (*φ*_1_ = 2°, *φ*_2_ = 12°) before the contrast agent injection (TR/TE = 4.04 ms/1.52 ms). The field of view and image grid was identical to the DCE‐MRI acquisitions. The blood T_1_ relaxation time was assumed to be 1.67 s.^21^ To facilitate registration of PET/CT and DCE‐MRI, anatomical transversal T_2_‐weighted images were additionally acquired for each patient, using a T_2_‐weighted short tau inversion recovery (STIR) sequence. STIR images were reconstructed to 46 slices, matrix size 320 × 320, and voxel size of 0.7 × 0.7 × 4.8 mm^3^. All MRI data were acquired on a combined PET/MRI scanner with the standard 16 channel head/neck coil.

### 2.B Image resampling, registration, normalization, and tumor volume delineation

For the purpose of correlation analysis, DCE‐MRI data were downsampled and filtered, so that their resolution corresponded to the resolution of PET images. DCE‐MRI downsampling was performed by binning 4 × 4 voxels in XY plane, as MRI voxel size in XY plane was roughly four times smaller than the XY voxel size for PET images. Subsequently, a 5 mm 3D Gaussian filter was applied to the downsampled DCE‐MRI — the same kernel size as used during PET reconstruction.

In order to match the DCE‐MRI grids, CT image components of FMISO‐PET data (dynamic, 2 h p.i. and 4 h p.i. images) were registered to STIR images with a previously described deformable registration method.^22^ Briefly, registration was performed using the Elastix toolkit^23^ with a b‐spline parameterized transform and mutual information as similarity measure. The resulting deformation field was applied to the FMISO‐PET images, which were subsequently resampled to the image grid of the downsampled DCE‐MR images.

Static FMISO‐PET images at 4 h p.i. were transformed to the tumor‐to‐muscle ratio (TMR) maps. A region in the neck muscle was segmented and FMISO‐PET images were normalized accordingly.

Tumor volumes, as well as the possible tumor bed and lymph nodes, were delineated for each patient by an experienced radiation oncologist. Delineations were done on corresponding planning CTs with the aid of FDG PET/CT, and were transferred to the downsampled DCE‐MRI datasets by deformable image registration of the planning CT to the STIR images.

### 2.C Kinetic analysis

Kinetic analysis of FMISO‐PET and DCE‐MR images was performed on a voxel basis by using image‐derived input functions (IDIF). Dynamic FMISO‐PET images were analyzed with a two‐tissue compartment model with three rate constants.^24^ DCE‐MR images were analyzed with the extended Tofts model.^25^ Segmentation of common carotid arteries for input function extraction was done with the MITK software.^26^ Kinetic analysis was done with a custom‐developed program in Matlab (The Mathworks, Natick, MA, USA).

#### 2.C.1 FMISO‐PET kinetic analysis

After registering the FMISO‐PET images to the downsampled DCE‐MR image, the dynamic 40‐min FMISO‐PET image, 2 h p.i. image, and 4 h p.i. image were stacked into a single dynamic PET image that was subsequently used for kinetic analysis. Implementation of the model followed Eq. (1), where *C*_*D*_ *(t)* and *C*_*A*_ *(t)* are *diffusive* and *accumulative* compartment concentrations, *I*_*P*_ *(t)* is the input function that is the plasma time activity curve, *λ* is the^18^F radioisotope decay constant, and *S(t)* is the signal of the model‐predicted concentrations determined by the PET measurement.

(1) $$\begin{array}{cc} \frac{dC_{D}\left(t\right)}{dt} & =K_{1}I_{P}\left(t\right)-\left(k_{2}+k_{3}-\lambda \right)C_{D}\left(t\right)\\ & \frac{dC_{A}\left(t\right)}{dt}=k_{3}C_{D}\left(t\right)-\lambda C_{A}\left(t\right)\\ & S\left(t\right)=C_{D}\left(t\right)+C_{A}\left(t\right)+V_{b}I_{P}\left(t\right) \end{array}$$

Parameters *K*_*1*_, *k*_*2*_, and *k*_*3*_ are rate constants of the compartmental model. The *K*_*1*_ parameter is the FMISO transport rate constant into the tissue, the *k*_*2*_ parameter is a FMISO backflow rate constant, the *k*_*3*_ parameter is the rate constant of FMISO tracer binding in the cells, and the parameter *V*_*b*_ is the vascular fraction in the tissue. Additionally, the parameter *K*_*i*_ *= K*_*1*_ *k*_*3*_ */(k*_*2*_ *+k*_*3*_ *)* has been evaluated and used in the subsequent analysis, because this parameter quantifies the FMISO uptake rate into the tissue. The underlying assumption behind this model is that plasma‐specific FMISO activity and total blood‐specific activity are equal for the whole imaging period, which implies that there are no radiolabelled metabolites in the blood pool and that FMISO freely enters into the blood cells.

The IDIF for kinetic analysis was obtained by placing a ROI in the largest vascular structure in the field of view (common carotid artery) and taking the average time activity curve over this region. Model equations in Eq. (1) have been solved analytically and the PET measurement *S(t)* has been evaluated by using a cumulative representation of the IDIF^27^ and a variable delay *t*_*d*_ between the input function and tissue signal.

#### 2.C.2 DCE‐MRI kinetic analysis

For voxel‐based DCE‐MRI analysis, each voxel's signal–time curve was fitted with a model‐predicted DCE‐MRI signal–time curve that utilized the extended Tofts model^25^ and nonlinear mapping of the Gd‐DTPA concentration to the MRI signal.^28^ The extended Tofts model has the following parameters: *K* ^*trans*^ is the contrast agent transport rate constant to the tissue, *v*_*e*_ is the interstitial (or extracellular extravascular) volume, and *v*_*p*_ is the vascular fraction in the tissue. Transformation between the Gd‐DTPA concentration and the MRI signal has been established through the spatial map of intrinsic T_1_ relaxation time obtained from VIBE images,^29^ and the first time frame of DCE‐MR imaging, when no Gd‐DTPA is yet present in the tissue. The extended Tofts model has been evaluated in Fourier space,^30^ using an IDIF and a variable delay *t*_*d*_ between the IDIF and the tissue signal. The IDIF was derived for each patient from the DCE‐MR images of original resolution. The IDIF was generated by placing a ROI in the largest vascular structure in the field of view (common carotid artery), taking the average signal–time curve over this region and applying a nonlinear transformation between the MRI signal and Gd‐DTPA concentration. Parameters were estimated by fitting the model‐predicted MRI signal curve to actual MRI measurements. For optimization, a parameter set of [*K* ^*trans*^ *, v*_*e*_ *, v*_*p*_ *, t*_*d*_] has been varied, with parameters being constrained to a viable interval.

### 2.D Statistical analysis

Pearson's correlation coefficients were evaluated for each patient and all available pairwise combinations of parametric maps (intra‐ and intermodality) and FMISO TMR at 4 h p.i. Median and minimum and maximum correlations for the patient cohort were reported. In addition, correlation coefficients are reported for all patients and for those combinations of parametric or TMR maps that had median correlation coefficients above 0.3. Correlation coefficients for pairs of parametric maps were evaluated by including all the voxels belonging either to tumor, lymph node, or tumor bed. Correlation analysis was done using Matlab (The Mathworks, Natick, MA, USA).

## 3 Results

Patient characteristics are shown in Table 1. Two patients had a severely hypoxic tumor, defined as TMR well above the threshold of 1.4,^31^ and only one of them had a severely hypoxic lymph node. Others were slightly hypoxic to nonhypoxic.

**Table 1:** Patient characteristics

PT	Number of lesions		Maximum TMR		Volume of lesions (mL)		Hypoxic fraction (TMR > 1.4)	
	Primary tumor	Nodes + tumor bed	Primary tumor	Nodes + tumor bed	Primary tumor	Nodes + tumor bed	Primary tumor	Nodes + tumor bed
P1	1	2	1.8	1.2	17.8	5.3	0.21	0.00
P2	1	3 + 1	1.6	1.2	24.0	13.4	0.18	0.00
P3	1	6	1.5	1.0	34.8	12.6	0.01	0.00
P4	1	3	3.8	1.5	48.5	16.6	0.56	0.02
P5	1	1	2.3	3.4	11.6	9.5	0.72	0.81
P6	1	1	1.8	1.0	34.5	1.6	0.15	0.00

**Figure 1:**
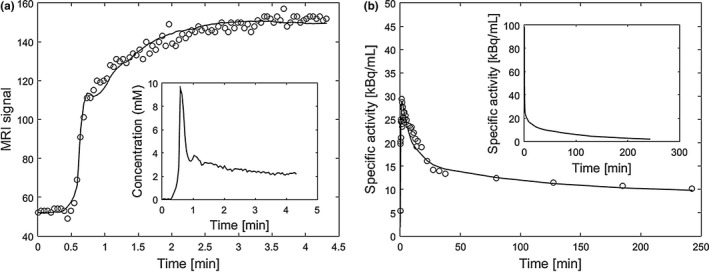
Exemplary DCE‐MRI signal–time curve and FMISO‐PET time activity curve for a random voxel inside the tumor with the corresponding arterial input functions (patient P1). (a) shows measured DCE‐MRI signal–time curve (circles), data fit according to the extended Tofts model (solid line), and the arterial input function (plot in the inset). (b) shows measured FMISO‐PET time activity curve (circles), data fit according to the compartmental model (solid line), and the arterial input function (plot in the inset).

Exemplary DCE‐MRI and FMISO‐PET signal–time curves for a random tumor voxel, together with model fits and corresponding arterial input functions are shown in Fig. 1. Mean parameter values and their standard deviations for transport rate constants and vasculature fractions are shown in Table 2.

**Table 2:** Mean parameter values and their standard deviations for transport rate constants and vasculature fractions

PT	FMISO‐PET *K*_*1*_		FMISO‐PET *V*_*b*_		DCE‐MRI *K* ^*trans*^		DCE‐MRI *v*_*p*_	
	Mean [mL/min/g]	Standard deviation [mL/min/g]	Mean	Standard deviation	Mean [mL/min/g]	Standard deviation [mL/min/g]	Mean	Standard deviation
P1	0.617	0.226	0.212	0.108	0.091	0.019	0.018	0.006
P2	0.677	0.282	0.181	0.078	0.129	0.096	0.057	0.026
P3	0.639	0.254	0.174	0.075	0.186	0.068	0.068	0.025
P4	0.640	0.261	0.048	0.040	0.145	0.050	0.023	0.009
P5	0.523	0.274	0.236	0.074	0.263	0.062	0.094	0.033
P6	0.443	0.268	0.195	0.126	0.118	0.062	0.107	0.058

The median correlation coefficients for DCE‐MRI kinetic parameters and FMISO kinetic parameters or TMR, respectively, varied between −0.05 and 0.71. The highest median correlation of r = 0.71 was found for the pair *V*_*b*_ *−v*_*p*_, while the *K*_*1*_ *−K* ^*trans*^ median correlation was r = 0.45. The *K*_*1*_ *−v*_*p*_ median correlation was r = 0.42 and *K*_*i*_ *−K* ^*trans*^ median correlation was r = 0.32. The TMR*‐K*_*1*_ and TMR*‐K* ^*trans*^ median correlations were r = 0.52 and r = 0.46, respectively. The median, minimum, and maximum correlation coefficients between the DCE‐MRI kinetic parameters and FMISO kinetic parameters or TMR are summarized in Table 3.

**Table 3:** Correlations between the FMISO and DCE‐MRI kinetic parameters. Median correlation coefficients are reported in the lower left triangle, and minimum/maximum in the upper right triangle

		FMISO kinetic analysis and TMR						Extended Tofts model		
		*K*_*1*_	*V*_*d*_	*k*_*3*_	*V*_*b*_	*K*_*i*_	TMR	*K* ^*trans*^	*v*_*e*_	*v*_*p*_
FMISO kinetic analysis and TMR	*K*_*1*_	1.00	0.29 0.74	−0.39 0.08	−0.01 0.51	−0.01 0.93	−0.17 0.59	0.27 0.59	−0.40 0.27	0.14 0.55
	*V*_*d*_	0.40	1.00	−0.23 0.26	−0.67 0.13	−0.09 0.57	0.26 0.71	0.09 0.62	0.03 0.78	−0.49 0.29
	*k*_*3*_	−0.11	−0.14	1.00	−0.12 0.24	−0.27 0.71	0.36 0.87	−0.27 0.35	−0.20 0.06	−0.16 0.22
	*V*_*b*_	0.21	−0.04	0.03	1.00	−0.13 0.34	−0.14 0.62	0.11 0.56	−0.41 0.02	0.42 0.74
	*K*_*i*_	0.32	0.11	0.50	0.22	1.00	−0.05 0.61	−0.20 0.45	−0.37 0.17	0.03 0.38
	TMR	0.52	0.67	0.44	0.27	0.51	1.00	−0.12 0.59	−0.13 0.60	−0.08 0.44
Extended Tofts model	*K* ^*trans*^	0.45	0.22	0.05	0.22	0.32	0.46	1.00	−0.06 0.86	0.34 0.86
	*v*_*e*_	0.03	0.14	−0.05	−0.05	−0.05	0.03	0.37	1.00	−0.30 0.35
	*v*_*p*_	0.42	0.08	0.02	0.71	0.12	0.23	0.58	0.15	1.00

Table 4 shows the voxel‐wise correlation coefficients for pairs of FMISO uptake parameters and DCE‐MRI kinetic parameters for all individual patients and for parameter pairs with at least a modest correlation coefficient (r ≥ 0.3) in the median across all patients.

**Table 4:** Correlations of FMISO and DCE‐MRI pairs of kinetic parameter for individual patients; shown are parameter pairs with at least modest correlation coefficient (r ≥ 0.3) in the median across all patients

	Parameter pair					
	*K*_*1*_ *−K* ^*trans*^	*K*_*1*_ *−v*_*p*_	*V*_*b*_ *−v*_*p*_	*K*_*i*_ *−K* ^*trans*^	TMR*‐K* ^*trans*^	TMR*‐K*_*1*_
P1	0.50	0.26	0.69	0.43	0.52	0.56
P2	0.27	0.40	0.72	−0.20	−0.09	0.59
P3	0.36	0.44	0.74	0.45	0.59	0.49
P4	0.59	0.55	0.46	0.25	0.40	0.19
P5	0.42	0.14	0.74	0.39	−0.12	−0.17
P6	0.48	0.49	0.42	0.08	0.53	0.56

**Figure 2:**
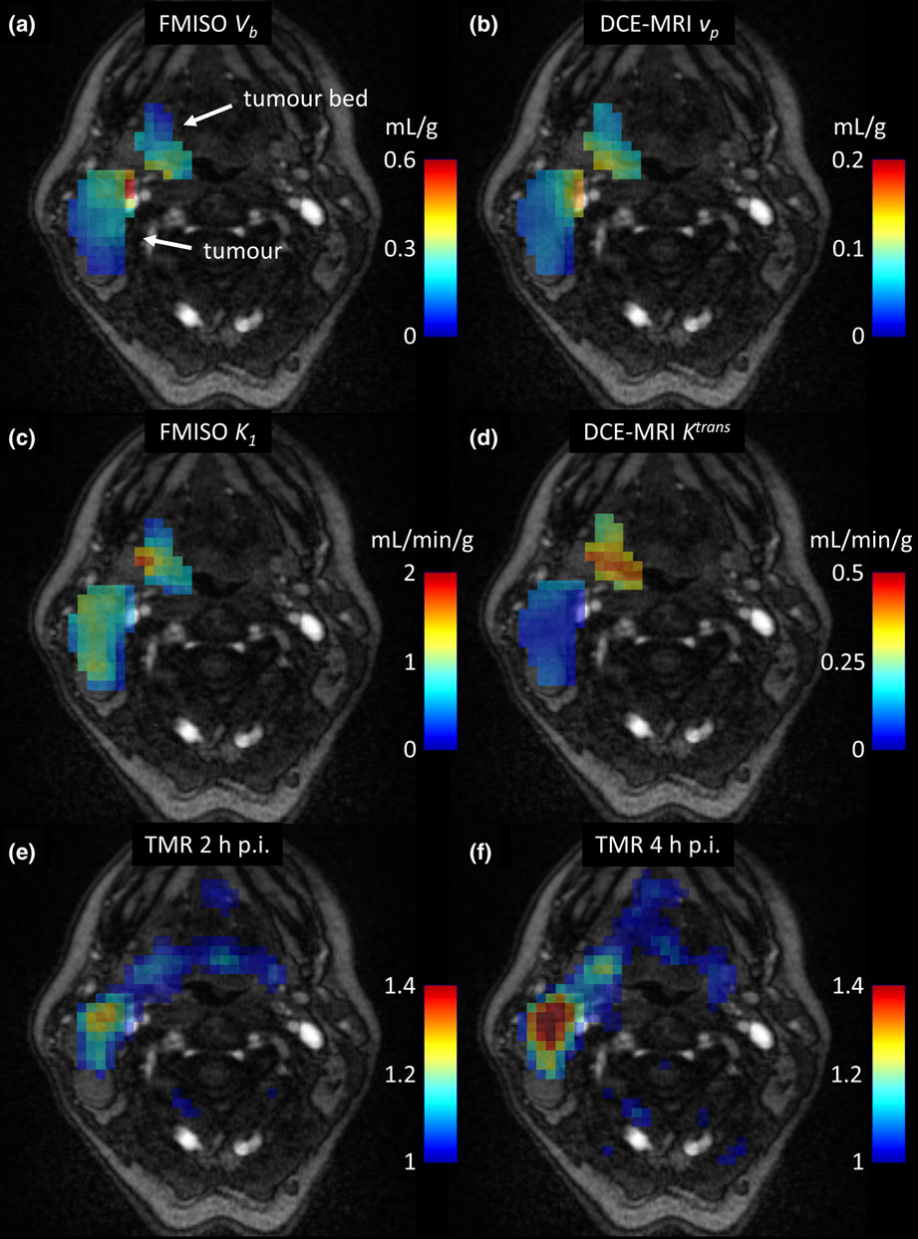
Contrast‐enhanced MRI images of the patient P2, overlaid with the FMISO *V*_*b*_ and DCE‐MRI *v*_*p*_ parametric images (a) and (b), FMISO *K*_*1*_ and DCE‐MRI *K* ^*trans*^ parametric images (c) and (d), and TMR maps at 2 h and 4 h post injection (e) and (f). [Color figure can be viewed at wileyonlinelibrary.com]

For one representative case (patient P2), the FMISO *V*_*b*_ and *K*_*1*_, DCE‐MRI *v*_*p*_ and *K* ^*trans*^ parametric images, and TMR maps at 2 h p.i. and 4 h p.i. are presented in Fig. 2. Visual patterns for the FMISO *V*_*b*_ and DCE‐MRI *v*_*p*_ parametric images are similar, as expected from a high correlation for the *V*_*b*_ *−v*_*p*_ pair. Areas of high *V*_*b*_ or *v*_*p*_ values correspond to the location of major vessels (visible on the underlying MRI with contrast agent). On the other hand, the tumor bed shows much higher DCE‐MRI *K* ^*trans*^ values than the tumor, whereas FMISO *K*_*1*_ has similar values in both, tumor and tumor bed. The hypoxic area (high TMR) is discordant to the area of high DCE‐MRI *K* ^*trans*^ and FMISO *K*_*1*_.

**Figure 3:**
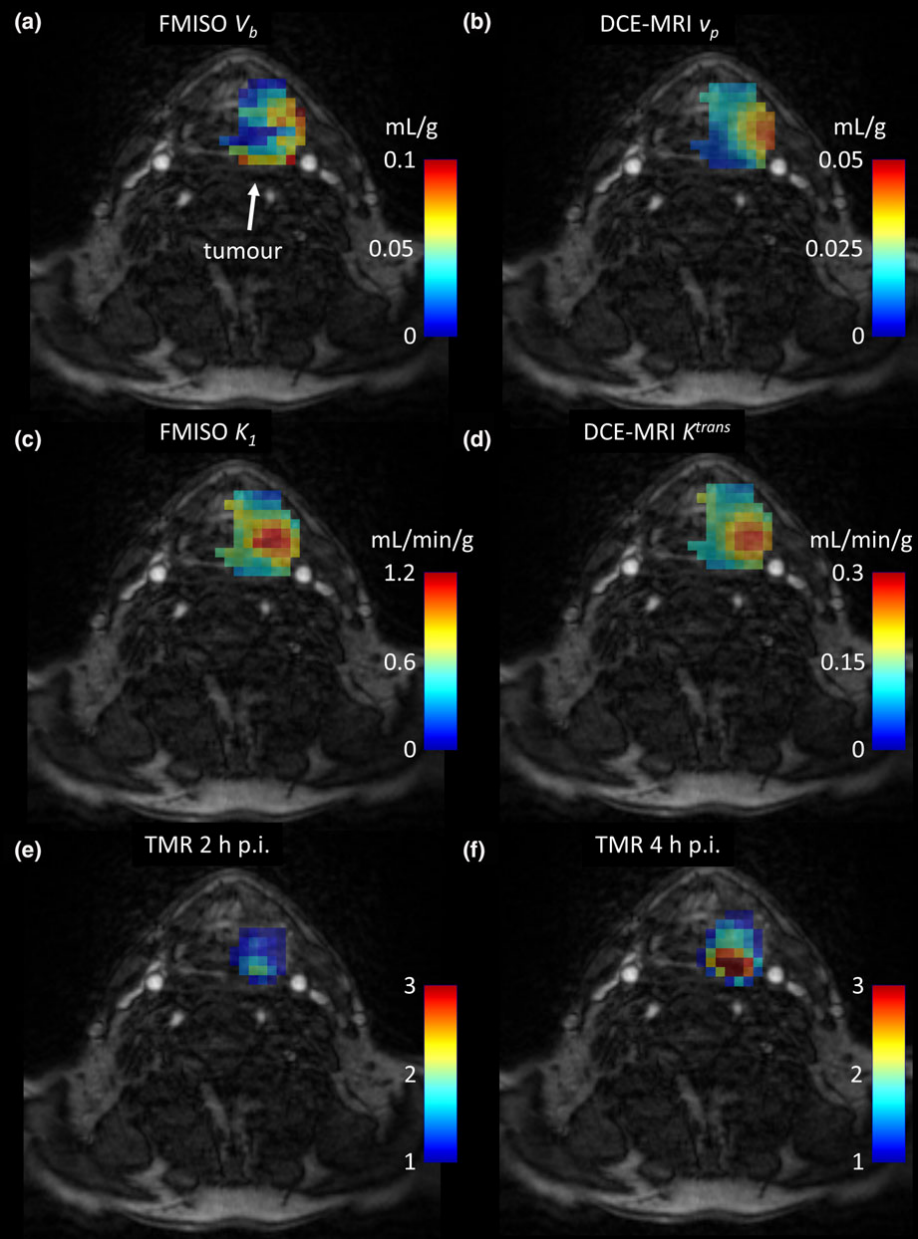
Contrast‐enhanced MRI images of the patient P4, overlaid with the FMISO *V*_*b*_ and DCE‐MRI *v*_*p*_ parametric images (a) and (b), FMISO *K*_*1*_ and DCE‐MRI *K* ^*trans*^ parametric images (c) and (d), and TMR maps at 2 h and 4 h post injection (e) and (f). [Color figure can be viewed at wileyonlinelibrary.com]

For another representative case (patient P4), the FMISO *V*_*b*_ and *K*_*1*_, and DCE‐MRI *v*_*p*_ and *K* ^*trans*^ parametric images are presented in Fig. 3. Visual patterns for the FMISO *V*_*b*_ and DCE‐MRI *v*_*p*_ parametric images are also similar, but there is one region inside that has low FMISO *V*_*b*_ values, but high DCE‐MRI *v*_*p*_ values. That must be the reason for the fairly low correlation of *V*_*b*_ *−v*_*p*_, but based on the visual pattern, it might be an artifact from kinetic analysis. Areas of high DCE‐MRI *K* ^*trans*^ and FMISO *K*_*1*_ are concordant, while the hypoxic area (high TMR) is discordant to the area of high DCE‐MRI *K* ^*trans*^ and FMISO *K*_*1*_.

**Figure 4:**
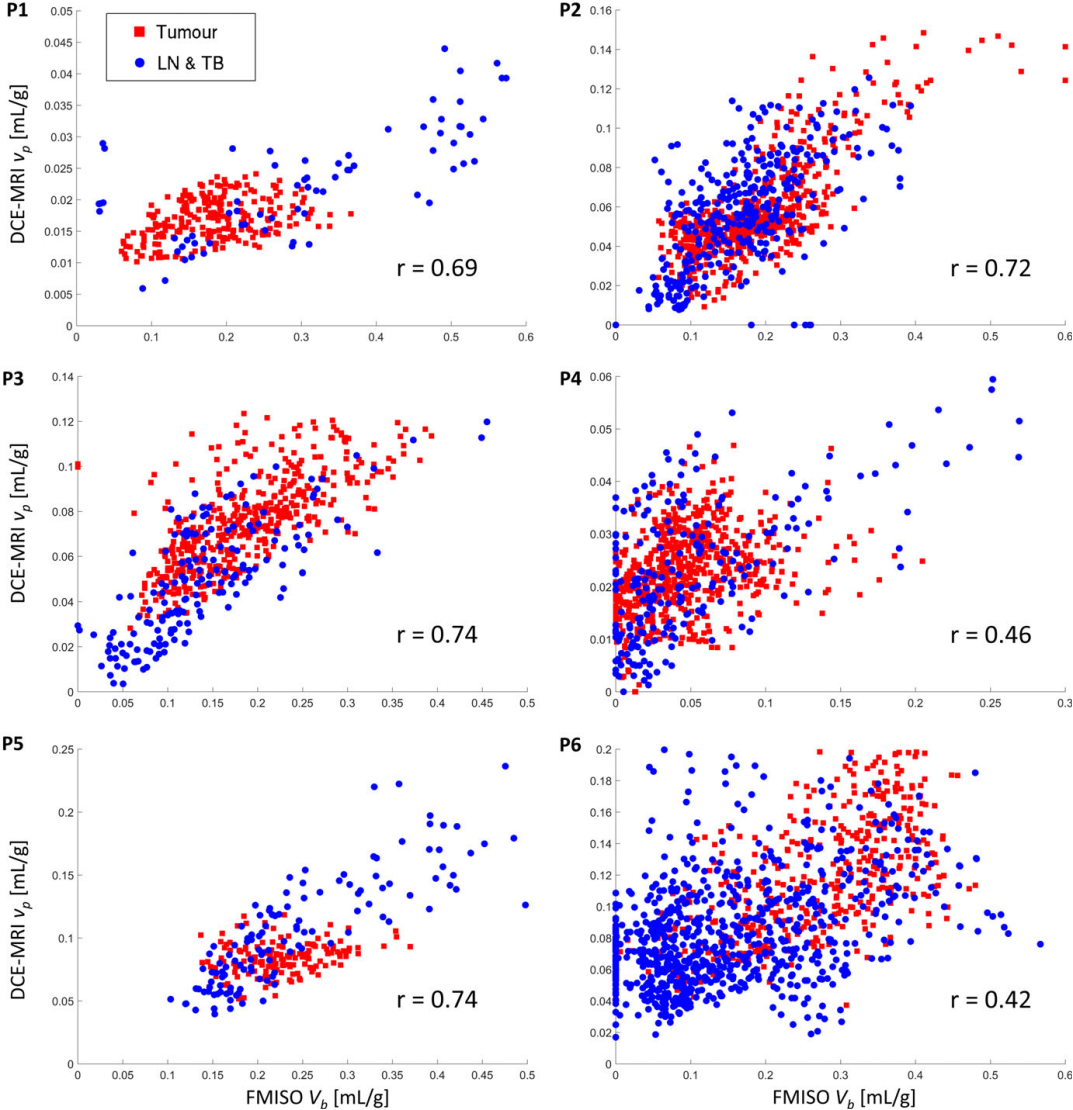
The *V*_*b*_ *−v*_*p*_ scatter plots for all six patients, with red squares and blue dots for tumor and lymph node or tumor bed voxels, respectively. [Color figure can be viewed at wileyonlinelibrary.com]

The *V*_*b*_ *−v*_*p*_ scatter plots for all six patients are shown in Fig. 4. Most plots show a distinct linear relation between the FMISO *V*_*b*_ and DCE‐MRI *v*_*p*_ parameters, as expected from high correlation coefficients. Patients P4 and P6 show more spread and less distinct linear relation, but no other relation is evident.

**Figure 5:**
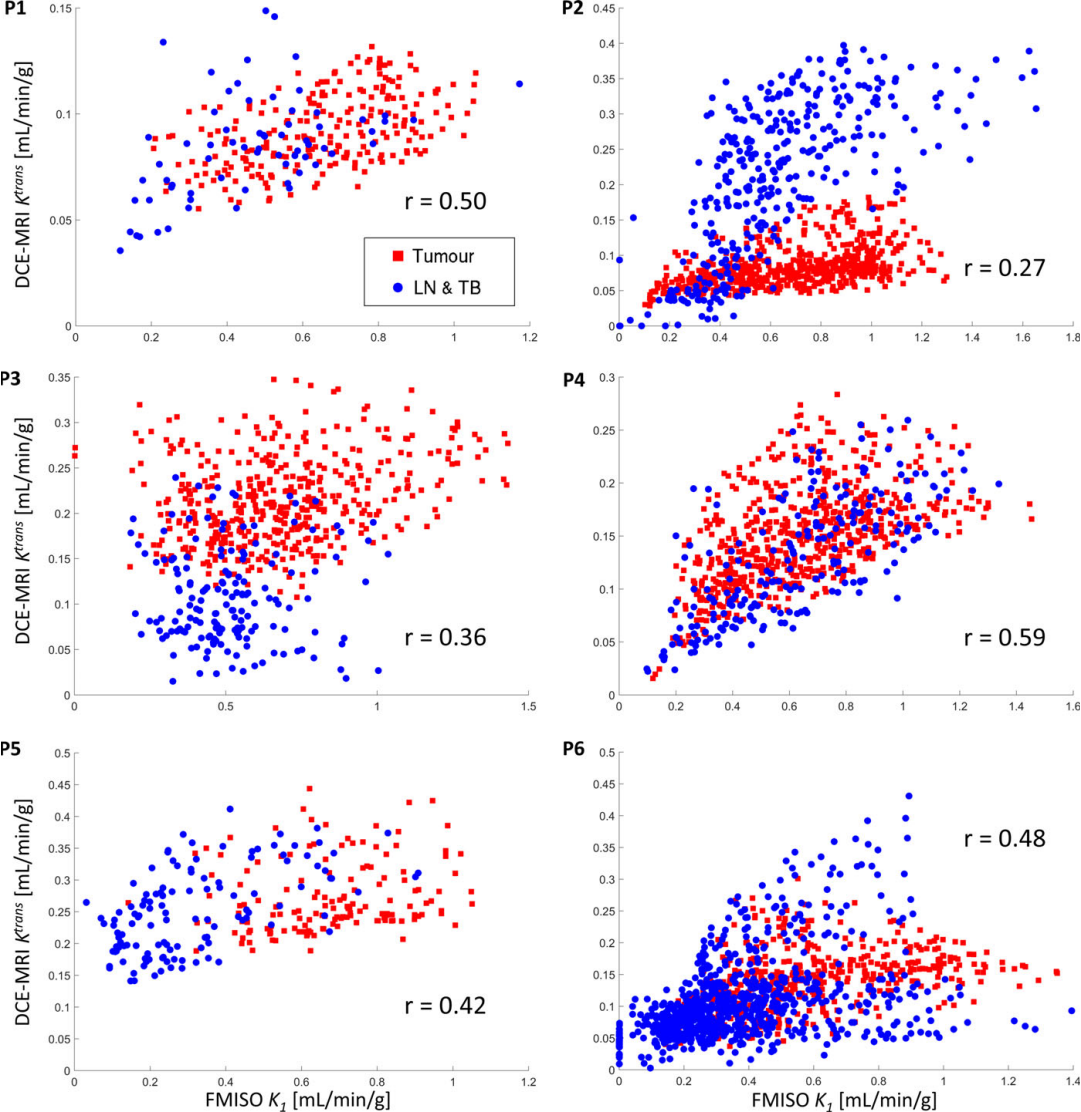
The *K*_*1*_ *−K* ^*trans*^ scatter plots for all six patients, with red squares and blue dots for tumor and lymph node or tumor bed voxels, respectively. [Color figure can be viewed at wileyonlinelibrary.com]

The *K*_1_ *−K* ^*trans*^ scatter plots for all six patients are shown in Fig. 5. Some scatter plots exhibit distinct *K*_1_ *−K* ^*trans*^ interrelation for tumor and other regions. The most notable example is patient P2, which was highlighted already in Fig. 2.

Scatter plots for TMR*‐K*_1_ and TMR*‐K* ^*trans*^ interrelations are shown in Supplement data.

## 4 Discussion

Our study was performed in head and neck cancer patients that were imaged dynamically with FMISO‐PET/CT and DCE‐MRI. Both image datasets were analyzed for kinetics as well as FMISO TMR at 4 h p.i., which allows a direct comparison of FMISO uptake parameters and DCE‐MRI kinetic parameters. We observed a high correlation of the vascular fractions measured with PET or MRI, respectively. Four of six patients showed *V*_*b*_ *−v*_*p*_ correlation coefficients around r = 0.7, which is a value for voxel‐wise correlations of two medical images that were acquired at different times, with patients in slightly different positions, and subsequently coregistered. Patient P4 that has only a moderate *V*_*b*_ *−v*_*p*_ correlation, presents with low *V*_*b*_ and *v*_*p*_ values, which implies lower accuracy of *V*_*b*_ and *v*_*p*_ estimates and therefore explains the lower correlation coefficient. Reasons for moderate *V*_*b*_ *−v*_*p*_ correlation in patient P6 are unknown.

Another potentially high correlation — the correlation between FMISO *K*_*1*_ and DCE‐MRI *K* ^*trans*^ parameters — was lower in median over all the patients. The different *K*_1_ *−K* ^*trans*^ relations in the main tumor and the other regions, as seen on scatter plots, indicate that those two kinetic parameters are different. The FMISO and Gd‐DTPA have much different molecular weight; consequently, the FMISO and Gd‐DTPA may have different permeability‐surface area products and thus different *K*_*1*_ or *K* ^*trans*^ transport parameters.

Correlations between the TMR and *K*_*1*_ or *K* ^*trans*^ transport parameters were also assessed and we found moderate positive correlation. Scatter plots show that for moderate TMR values, the TMR is actually higher for higher *K*_*1*_ kinetic parameter values, which might be due to the fact that FMISO uptake is driven mainly by FMISO inflow into the cells. However, high TMR values that are present in two patients are in voxels with low to moderate *K*_*1*_ kinetic parameter values. High TMR values are in hypoxic voxels, which are expected to be poorly perfused and therefore have low *K*_*1*_ kinetic parameter values. This agrees with a study by Donaldson et al.,^32^ who found significant negative correlations between the level of tumor hypoxia and perfusion and between the level of hypoxia and permeability.

Observed correlations between the FMISO uptake parameters and DCE‐MRI kinetic parameters indicate that DCE‐MR imaging provides similar information as the kinetic analysis of dynamic FMISO‐PET data. However, the information from both imaging modalities is not exactly the same. Therefore, FMISO‐PET/DCE‐MR multimodality imaging might be a good choice if the dynamic FMISO‐PET imaging is not possible, whereas the latter might still have its role in oncology research.

Although we have observed high correlations between the FMISO *V*_*b*_ and DCE‐MRI *v*_*p*_ kinetic parameters, we have to remark that these two parameters are far different in absolute values and the proportion factor is even not the same for all patients. However, this is most likely due to technical reasons, i.e., due to wrong scaling of both input functions that were derived from images and may be compromised with partial volume effects.

The presented work is impaired also by the small number of patients and considerable variation of results across the patients. Besides a low number of patients, the presented methodology is prone to the uncertainties that were introduced at kinetic analysis, image registration, and motion artifacts. Already small mismatches of only a single voxel can influence the correlation coefficient by 0.1 or 0.2.^33^ Another possible source of uncertainty for the correlation analysis is an ambiguous selection of the region, where voxel‐wise correlation is evaluated. In this work, we used regions that were supposed to receive any radiation dose during the RT planning, because we were interested in the interrelation between the FMISO and DCE‐MRI kinetic parameters for any region that could be targeted or evaluated in the process of RT treatment planning or assessment, so that more liberal inclusion of regions (i.e., primary tumor, tumor bed, lymph nodes) may be appropriate.

Despite these limitations, this study has provided some clinical evidence that may stimulate more research in FMISO‐PET/DCE‐MR multimodality imaging for the assessment of tumor microenvironment; in particular, tumor hypoxia and also tumor vasculature. In radiation oncology, information on tumor hypoxia can be used for biologically individualized radiotherapy (bio‐iRT),^34^, ^35^, ^36^ or identification of patients that may benefit from hypoxic radiosensitizers to supplement RT.^37^ Additional information on tumor vascular status could further characterize the tumors and can be convenient for therapy planning and response assessment in several hypoxia‐directed and angiogenesis‐directed therapies.

## 5 Conclusion

The results of this study indicate that the vascular fraction parameters obtained through DCE‐MRI kinetic analysis or FMISO kinetic analysis measure the same biological property. In addition, this study revealed poor correlation between other parameters. Potential reasons for poor correlation are different physiological processes that led to tracer distributions and physical processes involved in image acquisition, which need to be further explored. These results might be useful in the design of future clinical trials involving FMISO‐PET/DCE‐MR multimodality imaging for the assessment of tumor microenvironment.

## Conflict of Interest

There are no conflicts of interests.

## Supporting information


**Figure S1:** The TMR‐*K*_1_ scatter plots for all six patients, with red and blue dots for tumor and lymph node or tumor bed, respectively.
**Figure S2:** The TMR‐*K* ^*trans*^ scatter plots for all six patients, with red and blue dots for tumor and lymph node or tumor bed, respectively.
