# Supplementary material for: Comparison of DCE‐MRI kinetic parameters and FMISO‐PET uptake parameters in head and neck cancer patients
Source: Med Phys. 2017 Apr 20;44(6):2358–68. doi: 10.1002/mp.12228 (PMC5485084; doi:10.1002/mp.12228)
Supplement: Supplementary file 1 — Figure S1: The TMR‐K 1 scatter plots for all six patients, with red and blue dots for tumor and lymph node or tumor bed, respectively. Figure S2: The TMR‐K trans scatter plots for all six patients, with red and blue dots for tumor and lymph node or tumor bed, respectively. [file MP-44-2358-s001.pdf]

Supplement data

The TMR-K_1 and TMR-K^{trans} scatter plots

Scatter plots that exhibit TMR-K_1 interrelation are shown in Figure 1. The majority of voxels exhibit linear relation between the TMR and FMISO K_1, while the voxels corresponding to high TMR (only in patients P4 and P5) have low FMISO K_1.

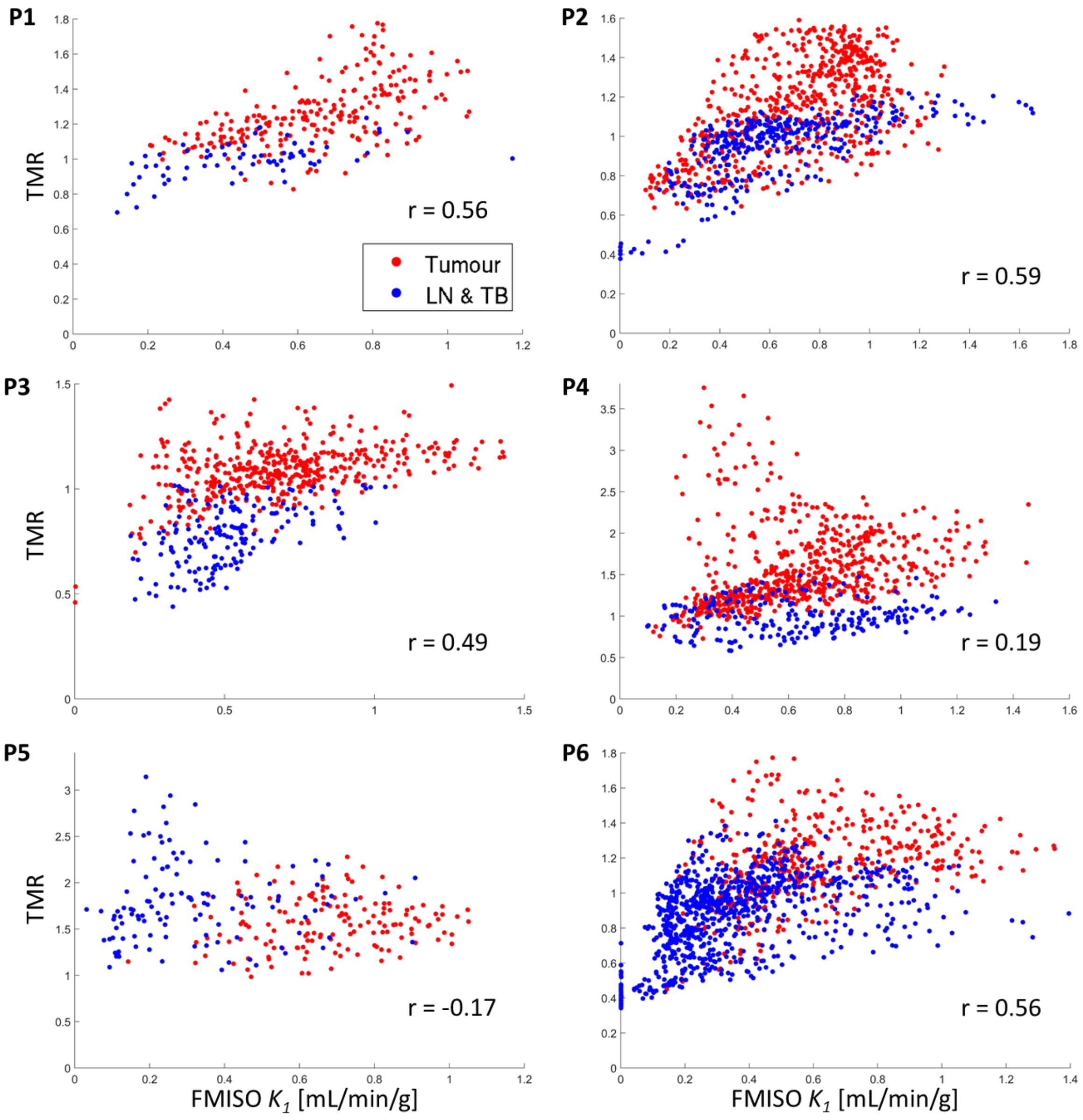

Figure 1: The TMR-K_1 scatter plots for all six patients, with red and blue dots for tumour and lymph node or tumour bed, respectively.

The TMR-K^{trans} scatter plots (Figure 2) appears to be different for each patient. For patient P3, for example, there is almost linear relation between the K^{trans} and TMR. Patients P1, P2 and P4 exhibit distinctive branching between tumour's voxels and voxels from lymph nodes or tumour bed. Patient P4 have some voxels with high TMR, but those voxels have intermediate to high K^{trans} values. In contrary to that, high TMR voxels in patients P5 and P6 have low or intermediate K^{trans} values.

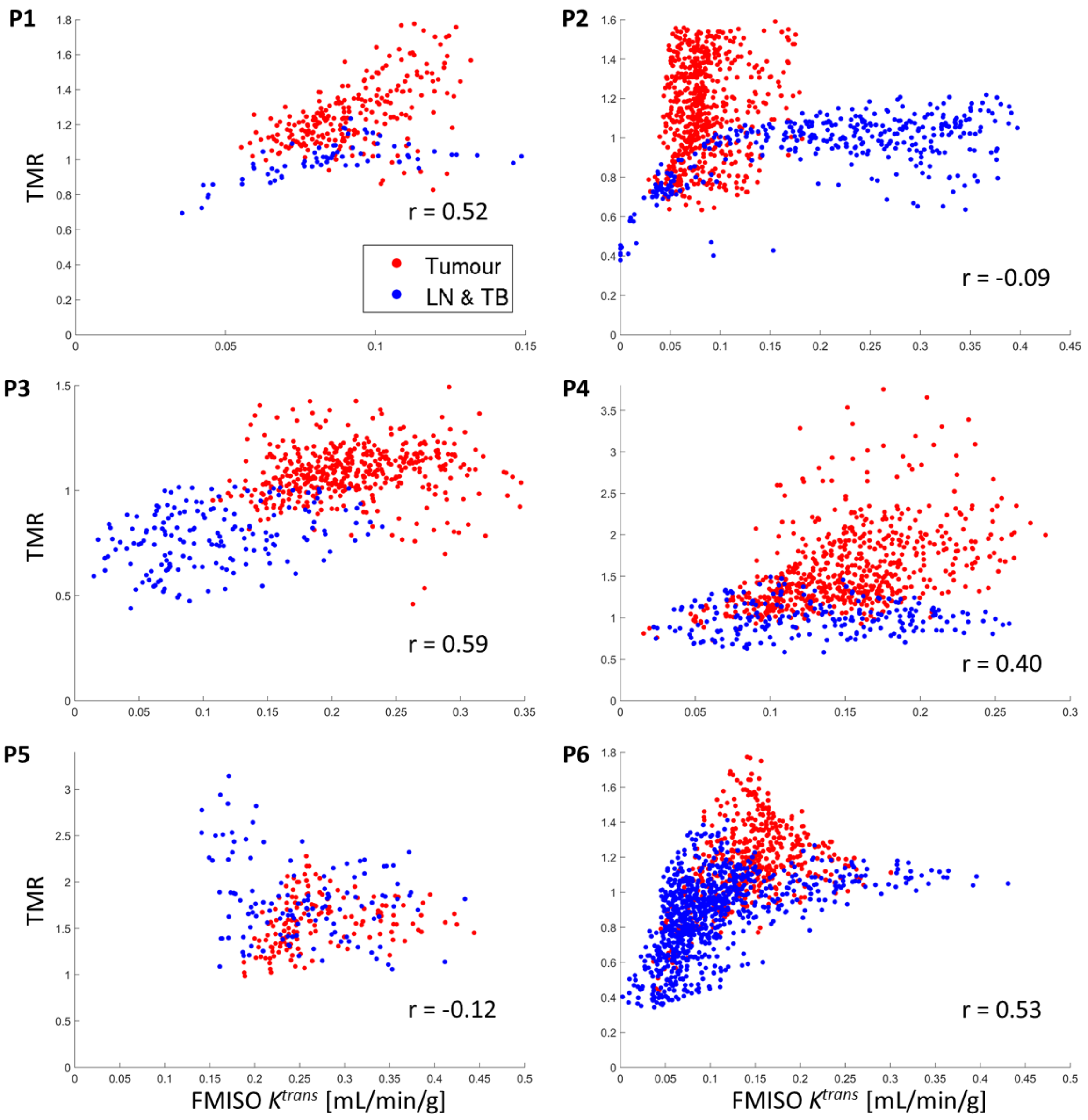

Figure 2: The TMR- K^{trans} scatter plots for all six patients, with red and blue dots for tumour and lymph node or tumour bed, respectively.
